# PACE-IT study protocol: a stepped wedge cluster randomised controlled trial evaluating the implementation of telehealth visual assessment in emergency care for people living in residential aged-care facilities

**DOI:** 10.1186/s12913-020-05539-1

**Published:** 2020-07-20

**Authors:** Carla Sunner, Michelle Therese Giles, Vicki Parker, Sophie Dilworth, Kamana Bantawa, Ashley Kable, Chris Oldmeadow, Maralyn Foureur

**Affiliations:** 1 Hunter New England Nursing and Midwifery Research Centre, James Fletcher Campus, 72 Watt Street, Newcastle, NSW 2300 Australia; 2 School of Nursing and Midwifery, University of Newcastle, University Drive, Callaghan, NSW 2308 Australia; 3 University of New England, Madgwick Drive, Armidale, NSW 2351 Australia; 4 Dementia Advisory Service Community Aged Care Services, Hunter New England Local Health District, Locked Bay 119, Wallsend, NSW 2287 Australia; 5 Hunter Medical Research Institute, Locked Bag 1000, Kookaburra Circuit, New Lambton, NSW 2305 Australia

**Keywords:** Agedcare, Telehealth, Telemedicine, Emergency department, Older person, Nursing home, Hospital avoidance, Visual telehealth

## Abstract

**Background:**

Transfer of residential aged-care facility (RACF) residents to Emergency Departments (ED) is common, risky and expensive. RACF residents who present to ED are more likely to have hospital readmissions, longer stays and face major risks related to hospital acquired complications. Aged Care Emergency services (ACE) is a nurse led, protocol- guided, telephone RACF/ED outreach model that has been shown to be effective in reducing hospitalisation and length of hospital stay for RACF residents in the Hunter New England Local Health District, New South Wales (NSW). The Partnerships in Aged-Care Emergency services using Interactive Telehealth (PACE-IT) project enhances ACE by incorporating interactive video assessment and consultation. The PACE-IT project’s primary aim is to assess whether augmentation of ACE services through the addition of protocol-guided interactive Visual Telehealth Consultation (VTC) for clinical decision-making, plus telephone follow-up, reduces RACF resident transfers to ED.

**Methods:**

A stepped-wedge cluster randomised controlled trial will be conducted. The intervention will be delivered sequentially to 8 clusters; each cluster comprises one ED and two RACFs in NSW, Australia.

The 16 RACFs in the study will be selected for order of implementation using a computer-generated randomisation sequence. A 2-step randomisation process will be undertaken, randomising the hospital EDs first and then randomising the RACFs aligned with each hospital.

The PACE-IT intervention comprises: an initial phone call by RACFs to the ACE service in the ED; the ACE service in ED responds with a protocol-guided VTC, a management plan agreed between all participants; an automated consultation summary letter to the General Practitioner and the RACF; a post VTC 24 h follow-up phone call to the RACF.

**Discussion:**

If shown to be effective, the intervention has the potential to improve the clinical care and quality of life for residents. Findings will provide high level evidence that will inform sustainable change and broad translation into practice across NSW. It will show how the change has been achieved and highlight success factors for scalability and sustainability. It will inform review of processes, the development of policy and guidelines that will integrate PACE-IT into existing service models in NSW.

**Trial registration:**

The trial is registered with the Australian New Zealand Clinical Trials Registry (Trial ID ACTR N12619001692123) 02/12/2020.)

## Background

People aged over 65 are the fastest growing age group in the world with numbers predicted to double between 2019 and 2050, when one in six people will be aged over 65. For the first time in history, by 2050, people aged over 65 will outnumber those in the 15–24 age bracket [1]. This will place increasing pressure on existing health services [1], in particular emergency departments (ED) and residential aged care facilities (RACF). In Australia, in the six years from June 2012 until June 2018, there has been a 12.2% increase in RACF places; an increase of over 2% per year [1]. In 2016 the USA had 1,347,600 people living in RACFs [2].

Residents from nursing homes or RACFs, as referred in this paper, present to ED with many co-morbidities exposing them to complex and invasive investigations, treatments and procedures, many of which may not add value to their care [3–5]. A visit to ED exposes residents to three times the risk of new, acute respiratory or gastrointestinal infection [6], possible harm, emotional stress, or “iatrogenic complications” such as falls, medication errors, pressure injuries, delirium [3, 7–9] and death. RACF residents are more likely to be readmitted to EDs and have longer ED and hospital stays [3, 7–9]. As a consequence of a 12 h stay in ED almost one in five patients aged over 65 were reported to develop delirium, increasing their length of stay (LOS) in hospital by approximately one week [10]. Up to 40% of RACF resident transfers are considered to ED are avoidable [8]. With up to 75% of RACF residents transferred to ED annually the cost implications are substantial [11] potentially $AUS12, 657,379 annually [12].

Nurse-led RACF/ED outreach models have been shown to be feasible, acceptable and cost effective [12] while decreasing ED presentations, waiting times, hospital admissions and LOS [7, 13–15]. Benefits of nurse-led RACF/ED outreach, together and similar hospital and aged-care partnership models, are well documented in the literature [4, 11, 13]. Such models have the ability to streamline care for the RACF resident to facilitate their navigation throughout the health system in a safe and timely manner [16]. Some models report positive outcomes in communication with improved clinical handover, information sharing and staff having enhanced confidence in resident care [17].

The Aged Care Emergency (ACE) service model of care provides clinical support to nurses in RACFs, enabling residents to be managed at the RACF thus avoiding transfer to an ED [7]. The ACE service is a nurse led RACF/ED outreach model in Hunter New England Local Health District (HNELHD) in New South Wales (NSW), Australia. The ACE/Agedcare Service Emergency Team (ASET) nurse located in the ED provides this outreach service. The key principles of the service are to improve the experience and quality of care of residents, with better management of acute symptoms [18] ensuring the resident is receiving the best care in a timely manner.

Although RACF/ED outreach models, such as the ACE service for RACF residents, have been shown to significantly reduce hospitalisation, they could further reduce avoidable transfers to ED [3, 4, 7, 14, 19]. Currently models are limited by variable acceptance and uptake [12, 14, 15], lack of trained staff in RACFs, high RACF staff turnover, unavailability of resources, poor bi-directional communication and the restrictions of telephone-only assessment [12, 14, 20–22]. Some models are informal, reliant on “a passionate ED physician” [16] to provide advice, placing restrictions on the timeliness of their availability at the time of the call.

The use of a visually augmented telehealth consultation has been recommended as one means by which to overcome many of these challenges [10]. An unpublished local pilot project established that the Visual Telehealth Consultation (VTC) is acceptable to staff and families, and reduces the disruption and distress associated with unnecessary transfer to hospital for residents and families. Telehealth is a well-established means of supporting the RACF resident by providing timely consultation with RACF staff, enabling high quality health care, which can reduce unnecessary hospitalisation [23]. However, evidence supporting the use of VTC in RACF/ED outreach models of care is limited in number [24, 25].

A Nurse-led RACF/ED outreach model implemented in conjunction with VTC capability has the potential for synergistic benefits. Qualitative studies identify that visual telehealth adds value to care, timeliness of care and fills gaps in service provision [26]. The need for better engagement with staff, residents and families has also been highlighted as central to the success of future initiatives [14, 27, 28]. The addition of the VTC alone has been reported to reduce ED presentations and hospitalisation of residents by up to 37% [25].

More studies are needed to understand which telemedicine tools and processes are most effective in improving outcomes for residents. Previous video-based models have been hampered by unavailable or unreliable internet and the need for expensive equipment [26]. No studies have examined the effectiveness of the nurse-led ACE model with the addition of VTC in reducing ED presentations from RACFs. Reviews and meta-syntheses of VTC in RACFs indicate limitations to studies, inconsistent outcome measures and the need for more large-scale implementation studies [25, 29]. The aim of the Partnerships in Aged-Care Emergency services using Interactive Telehealth (PACE-IT) project is to determine whether the introduction of VTC can further reduce overall transfers of residents to ED.

## Methods/design

### Aims

The three aims of the study are to;

1. assess whether the augmentation of ACE services through the addition of protocol guided interactive VTC for clinical decision-making, plus telephone follow-up, reduces RACF resident transfers to ED compared to usual care.
2. assess the acceptability of the model to RACF and ACE/ASET staff as well as any barriers and enablers to implementation.
3. explore the experience of the model from the perspectives of residents and family in relation to their level of involvement in decision making, the management plan, communication and outcomes.

### Hypothesis

The PACE-IT intervention will result in a 30% reduction in RACF resident transfers to ED compared with usual care.

**Primary outcome**: reduction of 30% in the rate of ED presentations from RACFs per 100 RACF beds.

**Secondary outcomes**:

Secondary outcomes:

- Presentation to ED within 48 h post VTC consultation to identify any adverse events
- ACE/ASET and RACF staff perceived barriers and enablers to implementation and sustainability at three months post intervention
- ACE/ASET and RACF staff acceptability and engagement three months post intervention; RACF staff perceptions of VTC usability survey within 48 h of participating in a VTC
- Resident and family experiences of participating in the intervention one-month post implementation
- Cost consequence analysis

### Design

**Fig. 1:**
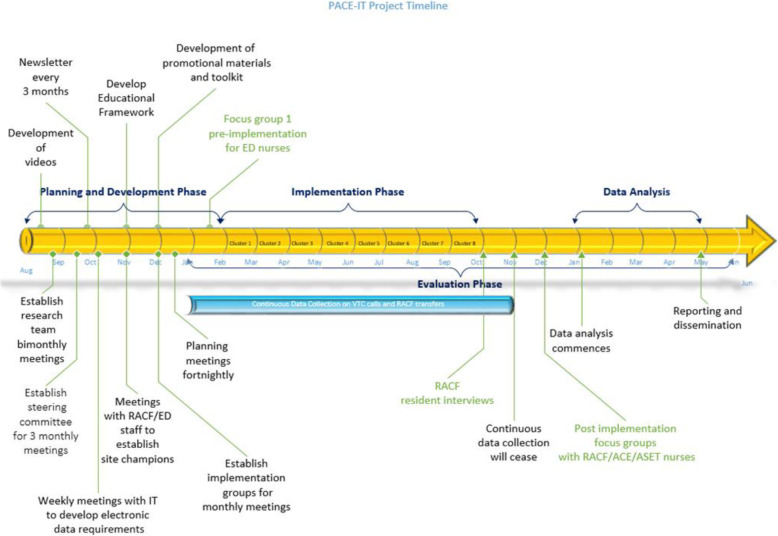
Overall study design and timeline

**Fig. 2:**
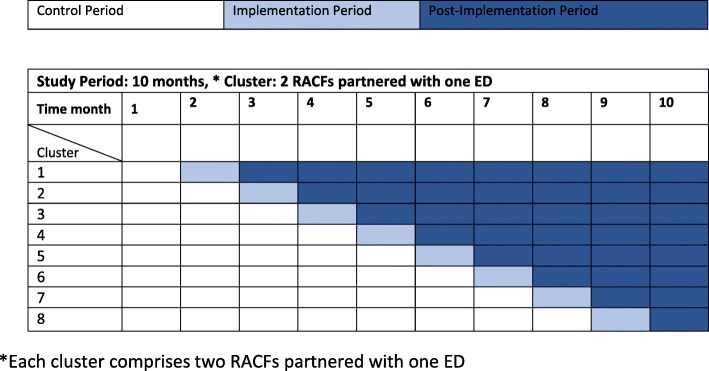
Implementation timeline for the stepped wedge cluster RCT study design

This implementation study uses a stepped-wedge cluster randomised controlled trial (RCT) design [30, 31] together with qualitative assessment of barriers and enablers to implementation and clinician and RACF resident/family acceptability of the PACE-IT intervention. Refer to Figs. 1 and 2. Whilst adhering to SPIRIT guidelines/methodology.

### Governance

Committee established for governance and overseeing of the project.

### Setting and participants

The intervention sites consist of EDs of four acute hospitals and 16 associated RACFs in two Local Health Districts (LHD) in NSW, Australia. The EDs have been selected for their metropolitan and rural locations; LHD A has two metropolitan and one rural ED and LHD B has one rural ED. Each cluster comprises one ED and two RACFs. Each selected ED has approximately 350 RACF beds, totalling 1435 beds across all 16 participating RACFs.

Residents and family members who have participated in a VTC call as a part of this intervention will be invited to participate in individual interviews in a private location within their RACF. RACF staff will be invited to complete an anonymous survey within 48 h of completing a VTC. RACF staff and aged care emergency nurses will be invited to participate in focus groups held in private locations within their facility or via videoconference.

Intervention participation: This is a cluster randomised control trial with an intervention that builds on an already embedded model of care (ACE model) [15], therefore residents requiring a VTC consultation will be in the participant group, but their written consent will not be sought. Consent has been obtained at an organisational level. Residents may choose to opt out of the VTC component and will experience the standard telephone consultation.

### Inclusion criteria

RACFs that have: a participating hospital ED from one of two participating LHDs; high rates of ED transfer (> 40 per 100 beds/annum); are VTC willing and able (Wi-Fi technology, mobile tablet, laptop or Workstation on Wheels available) agreed number of staff trained for VTC; high level support from the RACF organisational governance body.

EDs that have: ACE services and/or ASET nurses that provide an outreach service to RACFS.

RACF residents and family who experience the PACE-IT intervention.

RACF and ACE/ASET staff who have participated in the PACE-IT intervention.

### Intervention

The PACE-IT intervention provides an interactive VTC to enhance assessment and decision making augmenting the current ACE model of care. When a resident is unwell, and the GP is unavailable and there is a planned transfer to ED, the RACF staff member will initiate the following protocol:

1. **RACF nurse contacts ACE/ASET nurse** through a centralised 1300 phone number contacting the ACE/ASET nurse in the appropriate ED.
2. **Request for VTC** over the phone including information RACF staff member name, RACF facility name and the time of the proposed VTC, with a suggested time frame usually within 5–10 min.
3. **An interactive VTC is attended** by the ACE/ASET, RACF nurse, the resident and a family member if available.
4. **An ISBAR** [32] **handover** (introduction, situation, background, assessment, recommendation) is provide by the RACF nurse and the ACE/ASET nurse the details in the patients’ electronic medical record as an ED episode.
5. **A management plan is developed** through shared decision-making amongst the ACE/ASET nurse, the resident, RACF staff and any family members (if present in the consultation). A decision is made that the resident either remains in the RACF or is recommended to present to ED.
6. **A consultation summary** is auto generated and sent to the GP and RACF outlining the reason for the call and the outcome.
7. **A follow-up phone call** from the ACE/ASET nurse to the RACF will be undertaken within 24 h of the PACE-IT consultation, if the resident is not transferred to ED or admitted to hospital. Follow-up phone calls will identify what alternative non-hospital services were accessed, what treatment was delivered, and any adverse events. These data are also logged electronically in the ED patient management system (PMS).

NB. Usual care (ACE MoC) involves a phone call only with an ISBAR handover and an agreed management plan consistent with points 1, 4 and 5 above.

### Power and sample size calculation for the primary outcome

Local audit data identified 82 RACF ED presentations/100 beds annually. With the 16 RACFs contributing 1435 beds (Mean = 87) in this study, adopting the intervention in the sequence shown in Fig. 1, will have 80% power to detect a 35% relative reduction in ED presentations /100 beds annually (at 5% significance) assuming an intra-class correlation of 0.01.

### Randomisation

There will be four hospital EDs and 16 RACFs (four RACFs are aligned with each hospital ED). A 2-step randomisation process will be undertaken in Stata 14.1. Firstly, the four hospital EDs will be randomised, with each ED occurring twice, to create 8 clusters. Then the RACFs aligned with each hospital will be randomised in pairs, thus ensuring that each cluster has one hospital ED and two RACFs. The statistician was not blinded to the study sites during allocation.

The statistician was not blinded to the study sites during allocation.

### Recruitment

The ED recruitment will be made by contacting LHD facility executive to consent for their participation in the project. The RACFs will be recruited by contacting the appropriate executive requesting a signed letter of agreement that denotes their approval and consent participate. Following this recruitment strategy, we hope to achieve the required sample size.

#### Resident interviews

RACF staff will approach any resident who has participated in a VTC as part of the PACE-IT intervention within a month of the call, with an information letter, consent form and reply-paid envelope to enable them to return a signed consent to participate. The researcher will then contact the RACF to answer any questions the resident may have and to arrange a suitable date and time to conduct the interview. The participant must be able to provide informed consent to participate in interviews; this decision is made at the discretion of the RACF staff.

#### Focus groups

All ED and RACF members who have participated in a VTC as part of the PACE-IT intervention, will be invited to participate in a focus group via their work email. An information letter and consent form will be provided. Signed consents can be returned via email and consenting participants will be contacted to arrange a suitable date and time.

### Implementation strategy

Implementation strategies incorporating engagement, facilitation [33], education [34], resource development, resource deployment [30] and monitoring and feedback will be utilised to embed PACE-IT into participating facilities [35]. The Normalisation Process Theory (NPT) provides a framework to guide successful implementation and integration of complex interventions into routine practice [36]. NPT helps explain how interventions work through early implementation to beyond when the intervention becomes “embedded into routine practice and disappears from view” [36].

Prior to implementation commencing at each site, information sessions will be attended; video, progress newsletters and brochures will be circulated to inform stakeholders of the practice change involved in the intervention (e.g. general practitioners). Refer to Table 1.

**Table 1:** Implementation strategies

Strategy	Rationale	Delivered to and where	When/how often
**Engagement**			
Establish implementation groups	Increase awareness, identify barriers and develop context specific implementation strategies	RACF staff and ED ASET nurses	Establish 3 months before implementation and meet monthly before and during planning/intervention/implementation
**Education**			
ED visits by RACF staff	Increase awareness of residents’ ED transfer experiences	RACF staff and ED managers	Initial implementation of intervention and ongoing with change of RACF staff
RACF visits by ACE nurses and Telehealth Coordinators	Understand RACF context to enable implementation	ACE nurses at RACF sites	Every RACF at initial implementation
Education sessions on VTC and handover model	Increase RACF staff awareness of intervention	RACF staff	Initial implementation, ongoing with change of RACF staff
Staff training about video conference	Familiarise ED and RACF staff with video conference equipment used in intervention	RACF staff and ACE nurses at each ED	Initial implementation
**Resources**			
RACF Aged-Care Emergency Clinical Resource Manual	Guide ACE nurses in decision making for care of RACF residents	ACE nurses	Project start
Manual for VTC and handover model including video conferencing	Guide ACE nurses and RACF staff to normalise the VTC and handover via video conferencing	ACE nurses and RACFs	Project start
Establish video conferencing system	Familiarise ED and RACF staff with video conference equipment used in intervention	EDs and RACFs	Project start
Project information sheet and information videos	Inform staff at RACFs and EDs about proposed model of care	EDs and RACFs	Ongoing
**Compliance audits and feedback**			
Compliance audits and feedback	Monitor compliance and empower staff to continue with implementation strategies	Each site	Monthly from start of the implementation

### Data collection

Primary outcome data measuring RACF resident ED transfers and VTC calls will be collected monthly from the electronic PMS and will include information on demographics, presenting problem and call outcome (transfer to ED or alternative care pathway). All data will be stored securely in password protected electronic data bases and access will be restricted to selected members of the research team. All data will be de-identified to protect the confidentiality of the participants. All data will be cleaned and checked carefully prior to analysis.

Adverse events or unintended outcomes will be monitored by a post VTC 24 h follow-up phone call from the ACE/ASET nurse to the RACF as well as documenting any presentation of the RACF resident to ED within 48 h of the VTC. The follow-up phone call will ask six questions enquiring about: the resident’s condition, as well as confirming whether the consultation summary letter was received, providing any clarifying information if required and addressing any further concerns. The post VTC 24 h follow-up phone call will document the outcomes for any resident not transferred to ED as well as any alternative non-hospital service, outpatient service or treatment at the RACF. Data will be collected from the electronic PMS as a daily report on all VTC calls.

Pre-implementation video conference focus groups with four to eight ACE/ASET nurses inform potential barriers and enablers assisting with implementation/educational strategies.

Post-implementation focus groups (five) will be held with ACE/ASET nurses and RACF staff who participated in VTCs to identify barriers and enablers to uptake and ongoing sustainability and perceived benefits of the VTC interaction. These focus groups will be conducted three months post implementation.

Engagement, uptake and acceptability of the intervention for RACF staff will be measured using a 21-item electronic survey, sent via email to RACF staff the same day they participated in a VTC with the ACE/ASET nurse for completion within 48 h. The survey gathers information about RACF nurse demographics and VTC call details and includes Likert-scaled questions exploring the respondent’s experience related to accessibility, quality of the visual connection, engagement and usefulness of the VTC.

Acceptability and experiences of VTC for RACF resident and family will be obtained via 16–20 individual face to face or teleconference interviews with RACF residents and family members who were involved in a VTC as part of the PACE-IT intervention. Interviews with participants will be held within one month of their participation. Interview participants will be spread across all RACFS. Participants will be asked about their experience being involved in the VTC, specifically, their involvement in decision making, the management plan, communication and outcomes.

An external steering committee independent from the project investigators will monitor and discuss trial procedures. This committee consisting of members from the; NSW Ministry of Health, LHD executive, ethics, aged care, ED, statistician, RACF executive meeting every 3 months as per the protocol. This committee has the governance to stop the research if they recognise potential negative impacts on the well-being of participants, the committee will monitor adverse events and data discrepancies and will establish trial stopping rules as per the terms of reference. The senior researcher can convene a meeting with the committee at any time to review any unforeseen issues outside the 3 monthly scheduled meetings.

Missing data will be monitored each month and will be addressed on an ongoing basis as the project progresses. All noticeable omissions will be discussed at monthly meetings with the ACE/ASET staff and ongoing education will be carried out.

### Data analysis

Primary outcome, ED presentation data will be analysed to identify presentations involving transfer from the participating RACFs. The ED presentation data will be collected for each RACF per month, and the rate of ED presentations (per RACF beds) will be compared between intervention and control periods using a generalised linear mixed effects regression model (Poisson or negative binomial with a log link). The model will include; number of ED presentations in that period as the outcome variable; fixed effects for step and period; random effect for site; and the log number of RACF beds per facility as an offset term. The study is designed so that the onset of winter will be approximately the mid-point of the study, such that the aggregated control and intervention time periods across steps will involve equal amounts of winter/non-winter time (periods 4, 5 and 6, Fig. 1), thus accounting for seasonal variations during the “flu” season. The fixed effect for step will control for a common underlying secular trend across all clusters [31]. Data which is missing from the routinely collected datasets will be imputed for analysis.

Secondary outcomes will be quantitatively analysed with a descriptive summary collected from focus groups, interviews and surveys. Focus group and interview transcripts will be coded, categorised and themed using low-level interpretation to provide a narrative overview of staff experience [37]. Survey data analysis will provide descriptive statistics regarding staff, resident and family satisfaction with VTC and Information Technology (IT) processes.

A cost-consequence analysis (CCA) will be undertaken from the perspective of health services. The CCA will include intervention costs for labour, materials, overheads, travel, promotional materials and video production. The analysis will also capture downstream costs/costs-avoided through changes in ED presentations and readmissions. If results show evidence of intervention effectiveness, a budget impact analysis will be prepared, showing anticipated costs and outcomes over an annual health service budget cycle, with projections for three to five years.

### Dissemination plans

Communication of findings from the primary and secondary outcomes will be disseminated via peer-reviewed publications, conference presentations and local forums and will also be reported to the funding body, the ‘NSW Ministry of Health’. Findings will also be presented to the participating RACFs and at ACE interagency and implementation meetings (these will be held monthly throughout the project) and via regular PACE-IT newsletters.

The content of this research will help inform the review of processes, the development of policy and guidelines that will integrate PACE-IT into existing service models. The findings of this study will produce knowledge that will be sustained and spread through the stake holder network established as part of the project.

## Discussion

Currently there is limited rigorous evidence regarding nurse-led integrated models of care for assessment and treatment of acutely unwell RACF residents. If successful, this project will produce robust evidence regarding the effectiveness of a nurse-led interactive visual Telehealth integrated model of RACF/ED outreach care for RACFs. Evidence from this study will inform the design and delivery of a better connected and integrated health care system, supporting hospital avoidance and patient care delivered in the right place at the right time by the most appropriate healthcare provider [38].

When the PACE-IT model of care is implemented and translated into clinical practice it will result in reduced ED activity and reduced inappropriate use of the NSW Ambulance service and ED services for 12,622 older people currently residing in RACFs in LHDs A and B.

If scaled across NSW, the ACE/ASET VTC will benefit 68,967 such residents, potentially avoiding 15,000 avoidable presentations to ED per year.

The strength of the PACE-IT model is its potential for scalability and sustainability, with the advantage of utilising and enhancing existing resources and infrastructure like ACE/ASET nurses and the ACE model of care. There will be opportunity for the study partners to develop a strategy for rollout of the intervention more broadly in NSW. The participating EDs in the project will in turn be able to further extend PACE-IT model of care to all RACFs with whom they accept RACF transfers from. The RACF regional managers will have an opportunity to champion implementation across RACFs within their umbrella organisations.

Resources developed during this study will be available to facilitate the scalability and wider implementation of PACE-IT. Resources include PACE-IT guidelines developed by the PACE-IT governance group, including all relevant NSW stakeholders (Ministry of Health/ Clinical Excellence Commission/LHD/Primary Health Network (PHN)/RACF/NSW Ambulance Service). The PACE-IT education toolkit can be produced as an on-line resource, accessible at any time, so no additional toolkit or staff education costs are incurred. Other resources (posters, information brochures, information videos) will be made available to supplement the guideline and adapted for local contexts.

PACE-IT will inform a review of processes, the development of policy and guidelines that will integrate PACE-IT into existing service models. Executive endorsement and distribution of the guidelines through established systems will ensure wide dissemination of knowledge and the protocol for RACFs/EDs to implement. The viability of having the service extend to24 hours per day and 7 days per week will be determined by cost consequence analysis findings from this study. This will in turn inform ED decision-makers of the potential benefit of adopting 24/7 VTC for RACF residents in the future.

Findings of this study will produce knowledge that will be spread throughout the stakeholder network established as part the project. PACE-IT has the potential to improve the clinical care and quality of life of the resident. It will provide high level evidence that will inform sustainable change and translation into practice across NSW.

## Supplementary information

**Additional file 1.** PACE-IT Survey.

## Data Availability

The study data will be made available via a web-based de-identified data file to protect the privacy of participants and organisations described in the study, data available by contacting the first author of this paper.

## Abbreviations

ACE: Aged care Emergency
ACI: Agency for Clinical Innovation
ASET: Agedcare Service in Emergency Team
CCA: Cost Consequence Analysis
ED: Emergency Department
GP: General Practitioner
HNELHD: Hunter New England Local Health District
JHH: John Hunter Hospital
LHD: Local Health District
LoS: Length of stay
MOH: Ministry of Health
NSW: New South Wales
NUM: Nursing Unit Manager
PACE-IT: Partnerships in Aged-Care Emergency services using Interactive Telehealth
PHN: Primary Health Network
PMS: Patient Management System
RACF: Residential Aged Care Facility
VTC: Visual Telehealth Consultation
Wi-Fi: Wireless fidelity
