# Supplementary material for: PACE-IT study protocol: a stepped wedge cluster randomised controlled trial evaluating the implementation of telehealth visual assessment in emergency care for people living in residential aged-care facilities
Source: BMC Health Serv Res. 2020 Jul 20;20:672. doi: 10.1186/s12913-020-05539-1 (PMC7372753; doi:10.1186/s12913-020-05539-1)

PACE-IT Survey

PACE-IT Research Project Staff Survey

You are invited to take part in a survey related to the Visual Telehealth Aged Care Emergency (ACE) call you had recently.

Participation in this survey is voluntary and all information provided is anonymous.

Your participation in this survey will assist our team to evaluate the Visual Telehealth ACE call process and inform improvement strategies.

The survey should take less than 5 minutes to complete.

PACE-IT Survey

Section 1. Demographic information

Please select the response that applies to you

1. Gender*
   - Male
   - Female
   - Prefer not to answer

2. Age*
   - 30 years or less
   - 31-40 years
   - 41-50 years
   - 51-60 years
   - Over 60 years

3. What is your main role at the RACF?*
   - Registered Nurse
   - Enrolled Nurse
   - Assistant in Nursing
   - Manager
   - Administration staff

4. Do you speak a language other than English at home?*
   - No
   - Yes

5. How well do you speak/write English?*
   - Very well
   - Well
   - Not well

PACE-IT Survey

Section 2. About the Visual Telehealth ACE call

Please select or enter the response that applies to you

6. I have attended the following Visual Telehealth ACE call training programs: [comprising - 1) face to face education session at the RACF, 2) a visit to ED]*

- Face to face education session
- ED site visit
- Both
- Neither

Your response indicates that you have had no Visual Telehealth ACE call training. If you would like training, please call Carla Sunner on 02 4024 6720. Or email Carla.Sunner@health.nsw.gov.au.

7. What was the length of your latest Visual Telehealth ACE call in minutes?*

The response must be in whole minutes (eg 1 hr 15 mins should be entered as 75).

The value must be between 5 and 90, inclusive.

8. Overall, how many times have you participated in the Visual Telehealth ACE call to discuss a resident's clinical issue?*

- First time
- 2-4 times
- 5-10 times
- more than 10 times

9. Which RACF were you working in when you participated in your most recent Visual Telehealth ACE call?*

-- Please Select --

PACE-IT Survey

Section 3. Technology, Quality, Accessibility and Adaptability

This is about your experiences using a Visual Telehealth ACE call.
Please respond to the following statements by rating your level of agreement with each.

10. **Technology and Quality***

	Strongly Agree	Agree	Neutral	Disagree	Strongly Disagree
1. The Visual Telehealth ACE call was easy to set up	◯	◯	◯	◯	◯
2. The Visual Telehealth ACE call was easy to use	◯	◯	◯	◯	◯
3. The quality of the Visual Telehealth ACE call was acceptable	◯	◯	◯	◯	◯

11. **Accessibility and Adaptability***

	Strongly Agree	Agree	Neutral	Disagree	Strongly Disagree
1. The Visual Telehealth ACE call was too time consuming compared to a normal ACE phone call	◯	◯	◯	◯	◯
2. I have received good support from our facility champion in the use of Visual Telehealth ACE calls	◯	◯	◯	◯	◯
3. The training I received in the use of Visual Telehealth ACE calls has been adequate	◯	◯	◯	◯	◯

Your response indicates that you have had a problem with one or more of the Technology, Quality, Accessibility or Adaptability aspects while using Visual Telehealth ACE calls. If you would like advice or assistance, please call Carla Sunner on 02 4924 6720. Or email Carla.Sunner@health.nsw.gov.au.

PACE-IT Survey

Section 4. Usefulness and Engagement

Please respond to the following statements by rating your level of agreement with each.

12. **Usefulness** *

	Strongly Agree	Agree	Neutral	Disagree	Strongly Disagree
1. The Visual Telehealth ACE call enhanced communication between myself, the resident/carers and the ED clinician	◌	◌	◌	◌	◌
2. I believe the Visual Telehealth ACE call provided a more person-centred approach for the resident	◌	◌	◌	◌	◌
3. I was satisfied with the agreed resident management/care plan generated from the Visual Telehealth ACE call	◌	◌	◌	◌	◌

13. Your response indicates that you have had a problem with the Usefulness of Visual Telehealth ACE calls.
We value your opinion and would like to help solve any problems you are experiencing.
Please enter comments about your experiences using Visual Telehealth ACE calls so we can address these issues.
Please follow the ISBAR framework for your response:
Introduction
Situation
Background
Assessment
Recommendation

14. **Engagement***

	Strongly Agree	Agree	Neutral	Disagree	Strongly Disagree
1. My overall Visual Telehealth ACE call experience was positive	◌	◌	◌	◌	◌
2. I will use the Visual Telehealth ACE call process again	◌	◌	◌	◌	◌
3. I will recommend the use of Visual Telehealth ACE calls to other people	◌	◌	◌	◌	◌

Your response indicates that you have experienced a problem using Visual Telehealth ACE calls. If you would like advice or assistance, please call Carla Sunner on 02 4924 6720. Or email Carla.Sunner@health.nsw.gov.au.

Comments

15. Please provide any comments or suggestions that can improve the use of the Visual Telehealth ACE call to manage the situation for residents.

If you have an issue or a matter that you would like to discuss further with us, please contact the PACE-IT Research Team on 02 4924 6720.

Michelle Giles
Manager, HNE Nursing and Midwifery Research Centre
Email: michelle.giles@health.nsw.gov.au
or
Carla Sunner
PACE-IT Project Manager
Email: Carla.sunner@health.nsw.gov.au

Thank you
PACE-IT Research team

Thank you for completing the survey.

Your answers will now be saved, by clicking Submit.

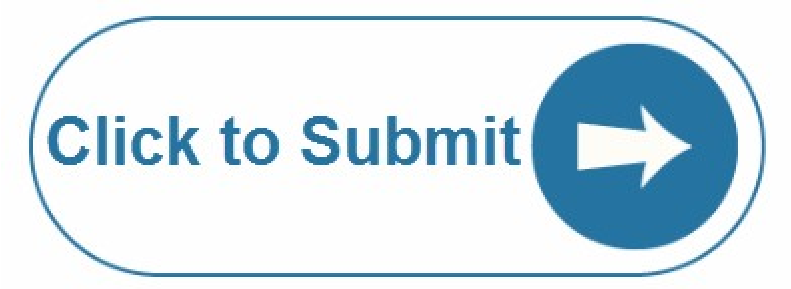

Supplement: Supplementary file 1 — Additional file 1. PACE-IT Survey. [file 12913_2020_5539_MOESM1_ESM.pdf]
